# “Cat Ladies” and “Mama’s Boys”: A Mixed-Methods Analysis of the Gendered Discrimination and Stereotypes of Single Women and Single Men

**DOI:** 10.1177/01461672231203123

**Published:** 2023-10-24

**Authors:** Hannah E. Dupuis, Yuthika U. Girme

**Affiliations:** 1 Simon Fraser University, Burnaby, British Columbia, Canada

**Keywords:** singlehood, gender, discrimination, stereotypes, singlism

## Abstract

Do single women and single men differ in their experiences of “singlism”? This mixed-methods research examined whether single women and single men report quantitative differences in amounts of singlehood-based discrimination and explored qualitative reports of stereotypic traits associated with single women and single men. We recruited Canadian and American single adults across two Prolific studies (total *N =* 286). The results demonstrated that single female and male participants did not differ in their personal discrimination, but female participants perceived single women to experience more discrimination than single men. Furthermore, qualitative analyses revealed four overlapping “archetypes” of single women and men including: *Professional* (“independent,” “hard-working”), *Carefree* (“free,” “fun”), *Heartless* (“selfish,” “promiscuous”), and *Loner* (“lonely,” “antisocial”). Overall, single women and men may experience similar stereotypes and discrimination, but there are also important nuances that highlight the need for more research at the intersection of gender and singlehood.

Despite singlehood becoming increasingly common (Statistics Canada, 2016; U.S. Census Bureau, 2021; United Nations, Department of Economic and Social Affairs, Population Division, 2019), single people endure negative stereotyping and discrimination (Budgeon, 2008; Byrne & Carr, 2005; Conley & Collins, 2002; DePaulo, 2007; DePaulo & Morris, 2005; Fisher & Sakaluk, 2020; Girme et al., 2022, 2023; Greitemeyer, 2009; Kaiser & Kashy, 2005; Morris et al., 2007; Uğurlu et al., 2021). “Singlism” is a term that describes both the oversimplification of beliefs about single people’s characteristics (i.e., stereotypes) and unfair or negative treatment (i.e., discrimination) due to not being in a romantic relationship (DePaulo & Morris, 2005). Yet, amid growing evidence that single people endure discrimination and stereotyping, there is some debate about whether single women experience more singlism than single men. For example, dozens of qualitative narratives from single women demonstrate that single women feel they are judged harshly due to their single status (Addie & Brownlow, 2014; Gui, 2020; Kawano et al., 2014; Moore & Radke, 2015; Pickens & Braun, 2018; Reynolds & Wetherell, 2003; Sandfield & Percy, 2003; Sharp & Ganong, 2011; Timonen & Doyle, 2014), with some scholars arguing that single women may experience more discrimination than single men (e.g., Kislev & Marsh, 2023; Lahad, 2013). Contrary to this, the few quantitative studies that exist have not found compelling differences between single women and single men in the amount of personal singlehood discrimination they experience (Conley & Collins, 2002; Greitemeyer, 2009; Morris et al., 2007). Of course, while single women and single men may both experience discrimination, the stereotypes of single women and single men may be qualitatively different and may hold implications for how women and men experience singlism.

By integrating qualitative and quantitative literature on gendered singlism, the current research uses a mixed-methods approach to provide the first research (that we are aware of) that directly assesses single women and single men’s beliefs about gendered stereotyping and discrimination. Specifically, in this research, we aim to (1) examine the amount of discrimination single female and single male participants (a) personally experience, and believe is directed toward (b) single people, (c) single women, and (d) single men. In addition, we aim to (2) qualitatively evaluate single female and single male participants’ perceptions of stereotypes about single women and single men.

## Singlehood Stereotypes and Discrimination

Single people experience stereotyping and discrimination for violating social norms that endorse romantic coupling as paramount. Instances of discrimination against single people often go unquestioned because of the pervasive notion that romantic bonds are integral for a meaningful, mature adulthood (Budgeon, 2008; Day et al., 2011; DePaulo, 2007; DePaulo & Morris, 2005; Fisher & Sakaluk, 2020; Girme et al., 2023; Kaiser & Kashy, 2005; Morris et al., 2007). Violating relationship norms leads to enduring singlism at the societal level (Budgeon, 2008; Day et al., 2011; DePaulo, 2007; DePaulo & Morris, 2005; Girme et al., 2023; Kaiser & Kashy, 2005; Morris et al., 2007). For example, married life comes with many privileges such as access to health and insurance benefits, tax breaks, and cheaper travel and living options that single people do not have access to (Budgeon, 2008; DePaulo, 2007; Girme et al., 2023; Kaiser & Kashy, 2005). Furthermore, research demonstrates that when group members are seen to have control over the devalued characteristics they possess (e.g., being single, higher body weight), negative attitudes toward them are seen as more acceptable than negative attitudes toward those who are discriminated against for things beyond their control (e.g., being a person of color; Crandall et al., 2002; see also Fisher & Sakaluk, 2020; Hunger et al., 2015). Thus, discrimination against singles tends to be overlooked because single status is seen as something people have control over; singles can exit their single status by finding a partner (Budgeon, 2008; DePaulo & Morris, 2005; Fisher & Sakaluk, 2020; Girme et al., 2023).

Single people are aware that they are treated badly due to their single relationship status and corroborate this with self-reported instances of discrimination (Byrne & Carr, 2005; Fisher & Sakaluk, 2020; Girme et al., 2022). For example, single people report equally high amounts of discrimination against their group as those from other systemically marginalized groups, such as members of the LGBTQ+ community (Fisher & Sakaluk, 2020). Singles also report lower well-being, which may be tied to experiencing more discrimination during day-to-day interactions compared with coupled people (Byrne & Carr, 2005; Fisher & Sakaluk, 2020), such as being told what is best for them, excluded from social events, treated them with pity, unfairness (Girme et al., 2022), or less respect, and receiving poorer service in restaurants (Byrne & Carr, 2005). This is indicative that beyond institutionalized forms of singlism, single people also report interpersonal discrimination because they do not have a romantic partner.

In addition to singlehood discrimination, single people are stereotyped more negatively than those in a romantic relationship. Single people are consistently rated as more miserable, lonelier, less trustworthy, and colder than coupled people (DePaulo & Morris, 2005; Greitemeyer, 2009; Hertel et al., 2007). Singles tend to be perceived by others as promiscuous and irresponsible (Hertel et al., 2007; Uğurlu et al., 2021), leading to assumptions that they are more likely to contract a sexually transmitted infection than coupled people (Conley & Collins, 2002). Similarly, rental agents rate single tenants as more likely to pay rent late, destroy the rental property, and break their leases than married tenants (Morris et al., 2007). Overall, singles are seen to possess less desirable personality traits, be less physically attractive, and less satisfied with their lives compared with those in romantic relationships (Greitemeyer, 2009; Hertel et al., 2007). These beliefs about singles reflect the stereotype that people are single because there is something wrong with them that undermines their partnering potential (DePaulo & Morris, 2005; Girme et al., 2023; Lahad, 2013; Moore & Radke, 2015; Sharp & Ganong, 2011; Zajicek & Koski, 2010). These stereotypical assumptions persist despite evidence that single and coupled people do not differ in personality traits or physical appearance (Greitemeyer, 2009).

## The Intersection of Gender and Singlehood

Experiences of singlism may vary depending on the other social identities single people hold—such as people’s gender. Gender has received the most attention in the qualitative singlehood literature because single women consistently report discrimination due to their single status (Addie & Brownlow, 2014; Gui, 2020; Kawano et al., 2014; Moore & Radke, 2015; Reynolds & Wetherell, 2003; Sandfield & Percy, 2003; Sharp & Ganong, 2011). For example, single women feel the need to assert that they are not desperate, explain their single identity, and believe they experience discrimination because they are seen as undesirable or defective (Addie & Brownlow, 2014; Moore & Radke, 2015; Pickens & Braun, 2018; Reynolds & Wetherell, 2003; Sandfield & Percy, 2003). Single women describe society’s perception that they are lonely or personally defective as the most salient source for their unhappiness (Gui, 2020; Moore & Radke, 2015; Pickens & Braun, 2018; Sandfield & Percy, 2003; Sharp & Ganong, 2011). Often, single women mention the struggles they face are unique to their gender because the very notion of womanhood is synonymous with tending to a home and childrearing (Addie & Brownlow, 2014; Gui, 2020; Kawano et al., 2014; Moore & Radke, 2015; Pickens & Braun, 2018; Sharp & Ganong, 2011). Although childless single women violate gender norms for not having a family to care for, gender norms for men dictate that they should be agentic and career driven (Gui, 2020; Jost & Kay, 2005; Kawano et al., 2014; Moore & Radke, 2015; Reuther, 1985; Rudman et al., 2012; Vandello et al., 2008). Thus, men who remain single to pursue career-related goals are not seen to be violating gender norms as overtly as women who remain single for similar reasons.

In contrast to the wealth of qualitative research on single women’s experiences, the qualitative research on single men has been sparse. Nonetheless, the extant qualitative research on single men’s experiences suggests that single men also experience singlism. A recent qualitative study of never married single men living in Poland found that single men reported negative stereotypes and discrimination similar to the narratives of single women (Mrozowicz-Wrońska et al., 2023). For example, single men described having their single identity questioned by others, violating masculine norms by not having a woman romantic partner, and being stereotyped as “womanizers” who are only interested in casual sexual relationships. Timonen and Doyle (2014) conducted a qualitative study on older single people living in Ireland and found that social norms kept single men from partnering unless they had the means to provide financially for a spouse, suggesting that singlism may be heightened for single men with lower socioeconomic status. The salience of coupling norms also appears in narratives of single men who express that people see them as immature (Mrozowicz-Wrońska et al., 2023) and incapable of caring for themselves (Timonen & Doyle, 2014; Zajicek & Koski, 2010). For example, one single man spoke of being pressured to hire a woman housekeeper because his family believed that if he were going to remain single, he would need the company and care of a woman in some capacity (Zajicek & Koski, 2010). The limited qualitative research on single men suggests that they also experience discrimination and stereotyping for being single, although this work has received much less attention.

Despite quantitative and qualitative research highlighting that single women and single men share experiences of stereotyping and discrimination, there is evidence that singlism may have gendered nuances for women and men. For example, Byrne and Carr (2005) found that while both single women and single men reported discrimination compared with married people, single men reported that others acted as if they were afraid of them and they were treated as if they were untrustworthy, while single women reported receiving poor service at restaurants and being called names. Similarly, quantitative research that looks at gender as a moderator of singlism highlights that while both single women and single men are rated as having worse personality traits compared with coupled people, single men are rated as more irresponsible (Morris et al., 2007; Uğurlu et al., 2021) and promiscuous (Conley & Collins, 2002) than single women. The contextual nature of singlism is further highlighted by the qualitative literature, in which younger single women speak of immense pressures to partner and have children (Addie & Brownlow, 2014; Gui, 2020; Moore & Radke, 2015), having their sexuality policed (Pickens & Braun, 2018; Reynolds & Wetherell, 2003), and being excluded from social gatherings with couples due to assumptions that they are deceitful and desperate (Reynolds & Wetherell, 2003)—themes that are absent in the qualitative studies on single men. Thus, while both single women and single men experience discrimination, stereotype content may reflect gendered nuances for single women and single men.

## The Current Research

This research provides novel social and theoretical insights to the singlehood literature. To date, extant literature provides inconsistent evidence about whether single women and single men differ in their perceptions of singlism. Although there have been many qualitative studies asking single women about their discriminatory experiences, far fewer qualitative studies have focused on single men’s experiences. No research to date (that we are aware of) has examined the amount of discrimination single women versus single men report experiencing due to their single status or perceive toward single women and single men as groups. Furthermore, much of the gendered nuances found in the literature appear to be driven by stereotypical assumptions about single women versus single men, yet no research has asked single people to describe stereotypes about their group. For the aforementioned reasons, this research is essential in shedding light on singlehood experiences at the intersection of gender and singlehood.

The goal of this research is to utilize a mixed-methods approach across two studies to (1) *quantitatively* examine the amount of discrimination single female and single male participants (a) personally experience, and perceive to be aimed toward (b) single people, (c) single woman, and (d) single men. This research will also (2) *qualitatively* examine the content of stereotypes about single women *and* single men. Note that “female participants” is used to describe participants who self-identified as “women” and “male participants” is used to describe participants who self-identified as men. These terms are used for clarity when describing study procedures and results; no information was obtained on participants’ sex assigned at birth.

Our pre-registered hypotheses are available on the Open Science Framework (OSF) for Study 1 (https://osf.io/9feg2) and Study 2 (https://osf.io/xy8ug). Our hypotheses were mostly the same across studies (exceptions are noted below), but we used clearer language in Study 2 regarding participant versus target gender, which is reflected in the hypotheses reported here. A detailed summary of the hypotheses is illustrated in Table 1.

**Table 1.** Summary of Pre-Registered Hypotheses and Results (Studies 1 and 2).

Pre-registered hypotheses			Hypothesis confirmed?	
Hypotheses	Study 1	Study 2	Study 1	Study 2
**H1**	Single women and single men will not differ in the amount of discrimination they report experiencing.	Single female participants and single male participants will not differ in the amount of discrimination they report personally experiencing.	✓	✓
**H2**	Single women and single men will not differ in the amount of discrimination they perceive toward singles as a group.	Single female participants will report more discrimination toward single people as a group than single male participants.	✕	✓
**H3**	Single men will report the same level of discrimination toward single women and single men.	Single male participants will report no differences in discrimination between single women as a group compared with single men as a group.	✓	✓
**H4**	Single women will report that single women experience more discrimination than single men.	Single female participants will report that single women as a group experience more discrimination than single men as a group.	✓	✓
**H5**	N/A	Single male participants will report more discrimination toward single men as a group than single female participants.	N/A	✓
**H6**	N/A	Single female participants will report more discrimination toward single women as a group than single male participants.	N/A	✓

*Note.* Study 2 had additional hypotheses pertaining to valence ratings of stereotypes that are not depicted here as they are not relevant to the current research. Please note the hypothesis numbers presented here match the order in the manuscript and may not correspond to the numbering in the pre-registration on Open Science Framework.

We hypothesized that:

**Hypothesis 1 (H1):** Single female participants and single male participants will not differ in the amount of discrimination they report personally experiencing.
**Hypothesis 2 (H2):** In Study 1, we hypothesized that single male and single female participants will not differ in the amount of discrimination they perceive toward single people as a group. However, based on the findings of Study 1, we conducted a confirmatory hypothesis in Study 2 such that we hypothesized that single female participants would report *more* discrimination toward single people as a group than single male participants.
**Hypothesis 3 (H3):** Single male participants will report no differences in discrimination toward single women as a group compared with single men as a group.
**Hypothesis 4 (H4):** Single female participants will report that single women as a group experience more discrimination than single men as a group.

In Study 2, we included secondary hypotheses for the contrast effects that assess in-group bias in perceptions of discrimination. Specifically, we also hypothesized that:

**Hypothesis 5 (H5):** Single male participants will report more discrimination toward single men as a group than single female participants.
**Hypothesis 6 (H6):** Single female participants will report more discrimination toward single women as a group than single male participants.

Across our analyses, we also conducted exploratory analyses that controlled for participants’ age, socioeconomic status, and parental status (that we included in our pre-registrations) to ensure that our findings remained unchanged when accounting for other intersectional identities that may impact singlehood stereotyping and discrimination. For example, having low socioeconomic status may lead some people to be involuntarily single as they cannot afford to provide for a family, making these singles particularly vulnerable to instances of negative treatment (Kislev & Marsh, 2023; Timonen & Doyle, 2014). Furthermore, Kaiser and Kashy (2005) argue that single people beyond the age of 36 are more likely to experience discrimination because they violate social norms more than younger singles. Discrimination may be especially salient for single people who remain single across the lifespan (Himawan et al., 2018; Mrozowicz-Wrońska et al., 2023; Timonen & Doyle, 2014). However, qualitative research highlights that (at least for childless single women) discrimination may be worse for singles during their childbearing years, as this is when they experience immense pressures to find a partner (Addie & Brownlow, 2014; Gui, 2020; Moore & Radke, 2015). For singles who have children, they may experience less interpersonal pressures than childless singles because others may assume that single parents are not voluntarily single. At the same time, however, single parents may experience more institutional forms of discrimination, such as economic instability (see Girme et al., 2023 for a review of singlehood nuances and parent status).

## Method

### Studies 1 and 2

We utilized a concurrent triangulation design for our mixed- methods analysis (see Creswell, 2013). This design was chosen because quantitative and qualitative data were collected simultaneously, and results were analyzed separately and compared afterwards. Study 1 aimed to assess whether there were differences in the amount of discrimination single female and single male participants reported and whether single female and single male participants reported distinct stereotype content about singles of their own gender. Study 2 aimed to replicate and extend these analyses by asking single female and single male participants to report stereotypic traits for *both* single women and single men. The procedures used in both studies are identical, and therefore presented jointly.

### Power Analyses

A priori power analyses using G*Power (3.1) software (Faul et al., 2007) indicated that to reach 95% power for a linear regression with three predictors, 119 participants were sufficient to detect a medium effect (*f* = 0.15). However, because Prolific dropout rates can range from 0% to 50%, we increased our sample size by 25% and aimed to recruit 150 participants.

### Participants

For Study 1, participants were 71 single women (including 2 trans-women) and 69 single men (*N* = 140)^1^ with an average age of 50.31 years (*SD* = 11.09; age range 36-81). The vast majority (91.4%) of participants identified as single and not dating while the remaining 8.6% were single and dating casually. In addition, 44.5% of our participants were never married, 24% were divorced once, 11% were divorced more than once, 15.8% had never been in a serious romantic relationship, and 4.8% were widowed. Participants reported being single for 11.39 years on average (*SD* = 12.74). Participants selected all ethnic/racial identities that applied, resulting in 82.9% White, 5.7% Black, 5.7% East Asian, 2.1% East Indian, 2.1% Indigenous, 2.1% Latin American, and 2.1% Middle Eastern identities. Most participants identified as heterosexual (85%), although 7.1% identified as bisexual, 4.3% as gay or lesbian, and 3.6% asexual. Most participants were not parents (62.1%), with the remaining 37.9% having 1 or more children.

For Study 2, participants were 70 single women and 76 single men (*N* = 146)^2^ with an average age of 49.77 years (*SD* = 11.48; age range 36-83). As in Study 1, the vast majority (89.7%) of participants identified as single and not dating while the remaining 10.3% were single and dating casually. Finally, 38.6% of the sample were never married, 30.7% were divorced once, 12.1% were divorced more than once, 14.3% had never been in a serious romantic relationship, and 4.3% were widowed. Participants reported being single for 13.75 years on average (*SD* = 14.57). Participants selected all ethnic/racial identities that applied, resulting in 80.8% White, 7.5% Black, 4.1% East Asian, 3.4% Latin American, 2.7% East Indian, and 1.4% Indigenous identities. Most participants identified as heterosexual (85.6%), although 5.5% identified as gay or lesbian, 4.1% as bisexual, 3.4% asexual, and 1.4% identified as pansexual. Most participants were not parents (64.4%), with the remaining 35.6% having 1 or more children.

### Procedure

Prolific workers who were registered as single (e.g., single, divorced, widowed, or never married), identified as women or men, over the age of 36, and residents of the United States or Canada were invited to participate in an online study about the experiences of single women and single men. Participants were invited to complete an online questionnaire that assessed their demographic information and their perceptions of singlehood-based discrimination and stereotypes, and other measures not germane to the current study. Participants were paid $6.24 CAD for completing the 20-minute questionnaire.

### Measures

#### Perceived Singlehood Discrimination

Similar to Taylor et al. (1990), participants were asked four questions regarding their perceptions of discrimination: “To what extent do you think people who are single experience discrimination?,” “To what extent do you think women who are single experience discrimination?,” “To what extent do you think men who are single experience discrimination?,” and “To what extent have you personally experienced discrimination for being single?.” The two questions asking about single women and single men were presented so that participants answered the question pertaining to their group (single women or single men) first. Responses were recorded on a 7-point scale (1 = *never*, 7 = *always*).

#### Perceived Singlehood Stereotypes

In Study 1, participants were asked two open-ended questions about the positive and negative stereotypes of single women or single men:

“What [negative/positive] characteristics do you believe people think single [women/men] possess? In other words, if you were to ask a random person to describe the [negative/positive], [bad/good] traits of a single [woman/man], what words might they come up with? Please list as many as you can think of.”

Single female participants were asked to provide stereotypes about single women, and single male participants were asked to provide stereotypes about single men.

In the qualitative coding analysis from Study 1, participants provided an average of 5 positive and 5 negative stereotype words. In Study 2, to make coding more efficient and reduce burden on participants reporting on both groups, participants were asked to provide up to 10 trait words to describe stereotypes of singles of their own gender, and up to 10 trait words to describe singles of the other gender:

“What characteristics do you believe people think single [women/men] possess? In other words, if you were to ask a random person to describe the traits of a single woman/man, what words (positive, neutral, or negative) might they come up with? Please list as many words as you can in the boxes below.”

## Results

A summary of all hypotheses and whether they were confirmed is presented in Table 1. Descriptive statistics for both Studies 1 and 2 appear in Table 2.

**Table 2.** Descriptive Statistics for All Measures (Studies 1 and 2).

	Female participants	Male participants
Measures	*M* (*SD*)	*M* (*SD*)
Study 1		
Personal discrimination	3.31 (1.36)	2.93 (1.38)
Discrimination against single people	4.20 (1.06)	3.54 (1.21)
Discrimination against single men	3.14 (1.21)	3.64 (1.33)
Discrimination against single women	4.75 (1.20)	3.78 (1.53)
Socioeconomic status	4.77 (1.64)	4.48 (1.94)
Participant age	52.76 (10.76)	47.78 (10.93)
Parental status^a^	0.51 (0.50)	0.25 (0.43)
Study 2		
Personal discrimination	3.17 (1.56)	2.72 (1.54)
Discrimination against single people	4.20 (0.97)	3.59 (1.23)
Discrimination against single men	3.31 (1.07)	3.82 (1.42)
Discrimination against single women	4.67 (1.05)	3.78 (1.27)
Socioeconomic status	4.59 (1.60)	4.38 (1.82)
Participant age	54.24 (12.18)	45.64 (9.08)
Parental status^a^	0.53 (0.50)	0.20 (0.40)

a In Study 1, 62.1% of participants had no children, 13.6% had one child, 8.6% had two children, 8.6% had three children, 6.4% had four children, and 0.7% had five children. In Study 2, 64% of participants had no children, 12.3% had one child, 10.3% had two children, 8.2% had three children, and 4.8% had four children. For the sake of our control analyses, we collapsed these data to indicate parental status where 0 = not a parent, 1 = parent.

### Do Single Female and Single Male Participants Differ in Their Perceived Personal Discrimination?

To test whether single female and single male participants differed in amounts of perceived personal discrimination, a regression analysis was conducted using SPSS Version 27. Specifically, perceived discrimination measures were regressed on participants’ gender (0 = women, 1 = men). Results are presented in Table 3. As predicted, in both Studies 1 and 2, there were no significant differences between single female and single male participants in the amount of personal discrimination they reported experiencing (H1).

**Table 3:** Regression Analyses Illustrating Participant Gender Predicting Reported Personal Discrimination and Discrimination Toward Single People as a Group (Studies 1 and 2).

	Study 1						Study 2					
	*b*	*t*	*p*	95% CI		*r*	*b*	*t*	*p*	95% CI		*r*
Outcome variable				Low	High					Low	High	
Personal discrimination	−0.38	−1.66	.10	−0.84	−0.08	.14	−0.45	−1.75	.08	−0.96	0.59	.14
Single group discrimination	−0.66	−3.44	<.001	−1.04	−0.28	.28	−0.61	−3.29	.001	−0.97	−0.24	.26

*Note.* Participant gender is coded 0 = women, 1 = men. Rosenthal and Rosnow’s (2007) formula: *r* = √(t2 / t2 + *df*). CI = confidence interval.

### Do Single Female and Single Male Participants Differ in Their Perceived Discrimination Toward Single People as a Group?

To test whether single female and single male participants differed in amounts of perceived discrimination toward single people as a group, a regression analysis was conducted. Specifically, perceived group discrimination measures were regressed on participants’ gender (0 = women, 1 = men). Results are presented in Table 3. Across both studies, single female participants reported significantly more perceived discrimination toward single people as a group compared with single male participants (contrary to H2 in Study 1 but supporting H2 in Study 2).

### Do Single Female and Single Male Participants Report Differences in Discrimination Toward Single Women versus Single Men?

Next, a repeated measures ANOVA^3^ was performed to compare single female and single male participants perceptions of the discrimination against single women versus single men. In Studies 1 and 2, the main effect of gender was not significant, suggesting that there were no significant differences in overall discrimination ratings for single female and single male participants (Study 1: *F*_(1,138)_ = 1.65, *p* = .20; Study 2: *F*_(1,144)_ = 1.30, *p* = .26). However, across both studies, the within subjects’ factors of target gender (single women vs. single men) was significant (Study 1: *F*_(1,138)_ = 44.91, *p* < .001; Study 2: *F*_(1,144)_ = 39.79, *p* < .001), which was represented by a higher-order interaction between Participant Gender × Target Gender, suggesting that the effect of participant gender on target gender discrimination ratings differed depending on whether ratings were for single women or single men (Study 1: *F*_(1,138)_ = 31.27, *p* < .001; Study 2: *F*_(1,144)_ = 44.70, *p* < .001).

**Figure 1.**
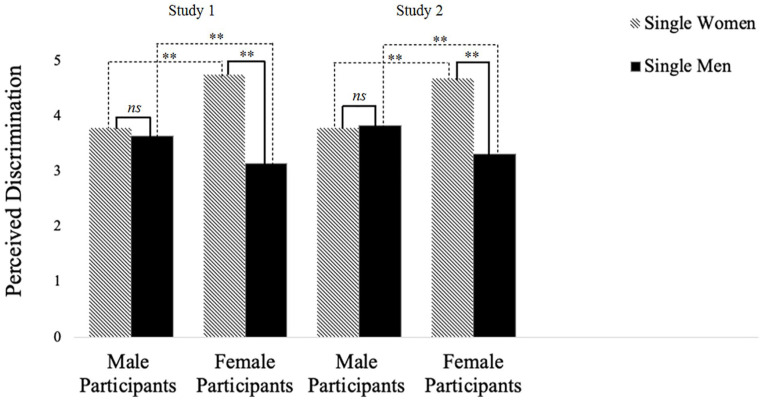
Mean Differences in Perceived Discrimination Against Single Women Versus Single Men as Rated by Female and Male Single Participants (Studies 1 and 2). *Note.* Solid contrast lines represent the results of paired samples *t*-tests across Study 1 (left) and Study 2 (right). Dashed contrast lines represent the results of the independent samples *t*-tests across Study 1 (left) and Study 2 (right). *ns* denotes non-significant differences. **p < .01.

Next, we conducted two paired-samples *t*-tests to test whether (a) single male participants and (b) single female participants reported different amounts of discrimination toward single women versus single men. Results are presented in Table 4 and Figure 1 (see solid contrast lines). As predicted, in Studies 1 and 2, male participants perceived similar amounts of discrimination directed toward single women and single men (H3). However, as predicted, female participants reported significantly higher amounts of discrimination toward single women as a group compared with single men as a group (H4).

**Table 4:** Paired Samples t-Tests Predicting Differences in Reported Discrimination Toward Single Women and Single Men.

	Study 1						Study 2					
	*M* diff *(W—M)*	*t*	*p*	95% CI		*d*	*M* diff *(W—M)*	*t*	*p*	95% CI		*d*
Participant gender				Low	High					Low	High	
Male participants	0.14	0.78	.44	−0.22	0.52	−0.94	−0.04	−0.28	.78	−0.32	0.24	−0.32
Female participants	1.61	8.76	<.001	1.24	1.97	1.04	1.36	8.83	<.001	1.05	1.66	1.06

*Note. W* denotes discrimination against single women, *M* denotes discrimination against single men. CI = confidence interval.

To test whether single female versus single male participants differed in discrimination reported toward (a) single women as a group and (b) single men as a group, we conducted two independent samples *t*-tests. Results are presented in Table 5 and Figure 1 (see dashed contrast lines). As predicted, in both Studies 1 and 2, single female participants reported significantly higher discrimination toward single women as a group compared with single male participants (H5 in Study 2). Furthermore, as predicted, single male participants reported significantly higher discrimination toward single men as a group compared with single female participants (H6 in Study 2).

**Table 5:** Independent Samples t-Tests Predicting Differences in Perceived Discrimination Against Single Women and Single Men.

	Study 1					Study 2				
	*t*	*p*	95% CI		*d*	*t*	*p*	95% CI		*d*
Outcome variable			Low	High				Low	High	
Perceived discrimination against single women	4.15	<.001	0.50	1.42	0.39	4.62	<.001	0.51	1.27	0.40
Perceived discrimination against single men	−2.31	.02	−0.92	−0.71	0.39	−2.39	.02	−0.91	−0.09	0.40

*Note.* The grouping variable was participant gender, which was coded 0 = women, 1 = men. CI = confidence interval.

### Control Analyses

We re-ran regression analyses from Table 3 to control for any distinct effects of singlism based on lower socioeconomic status, older age, or parental status (see Byrne & Carr, 2005; Girme et al., 2023; Kaiser & Kashy, 2005; Kislev & Marsh, 2023; Timonen & Doyle, 2014). Controlling for socio-economic status (SES; grand-mean centered)^4^ did not alter the non-significant effect of gender on perceived personal discrimination (Study 1: *t* = −1.60, *p* = .113; Study 2: *t* = −1.82, *p* = .07). Controlling for age (grand-mean centered) also did not alter the effect of gender on personal discrimination in Study 1 (*t* = −1.87, *p* = .064), but did reveal a significant gender effect in Study 2, suggesting that single female participants reported greater personal discrimination than single male participants when controlling for age (*t* = −2.57, *p* = .011). In addition, in Studies 1 and 2, the association between gender and personal discrimination became significant when controlling for parental status (0 = not a parent, 1 = parent), suggesting that single female participants reported experiencing greater personal discrimination compared with single male participants when controlling for parental status (Study 1: *t* = −2.40, *p* = .018; Study 2: *t* = −2.02, *p* = .045). However, further exploratory analyses did not reveal a Gender x Parental Status interaction, suggesting that there were no unique effects for single mothers (Study 1: *t* = −1.10, *p* = .27; Study 2: *t =* .66, *p =* 51.). Furthermore, controlling for age, parental status, and SES did not alter the significant effect of gender on perceived discrimination toward single people as a group (*t*s = −3.15 to −3.69, *p*s < .001).

In addition, repeated measures analyses were conducted to determine whether controlling for SES, age, or parental status altered results. The significant interaction between participant gender and target gender discrimination ratings remained significant controlling for SES, age, and parental status (*F*s = 26.01-44.03, *ps* < .001). Overall, the focal effects seemed robust when controlling for SES, age, and parental status (with some indication that women may experience more personal discrimination than men when controlling for parental status).

### Do Single Female and Single Male Participants Report Gendered Stereotypes About Single Women and Single Men?

The open-ended responses in both Studies 1 and 2 were examined by conducting a qualitative coding analysis (adapted from methodology outlined by Creswell & Poth, 2017). For Study 1, four research assistants blind to all research aims coded the qualitative responses. The coders were divided into pairs. Two coders read through and coded the *negative* stereotypes and two coders read through and coded the *positive* stereotypes provided by participants. For Study 2, two coders coded all the data. For both studies, coders used an Excel sheet with the open-ended responses to tally the trait words provided and organize them into synonymous “codes.” For example, if three participants wrote “lonely” and one wrote “lonesome,” that would be four tallies under the code “lonely.” After coding, the pairs of coders compared their frequencies and resolved any inconsistencies.

**Figure 2:**
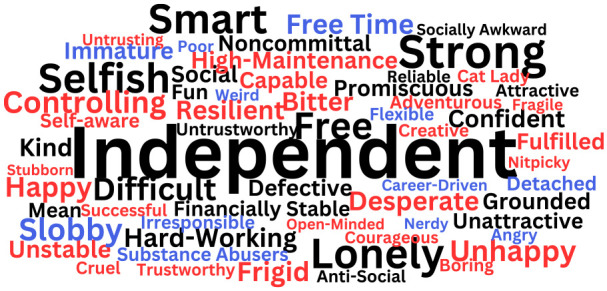
Shared and Unique Stereotypes of Single Women and Single Men (Study 1). *Note.* Study 1 had participants report stereotypes for singles of their own gender only. Black words reflect stereotypes used by single participants to describe both single women and single men; red words reflect stereotypes provided by single female participants to describe single women; blue words reflect stereotypes provided by single male participants to describe single men.

**Figure 3.**
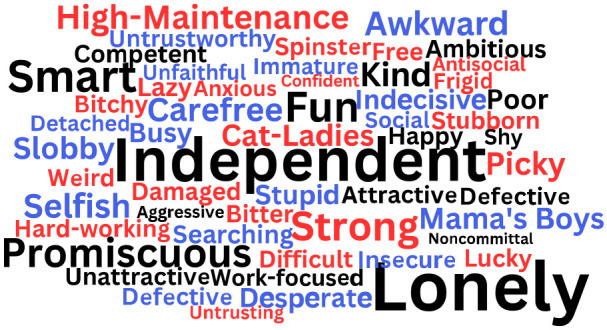
Stereotypes of Single Women and Single Men Provided by Single Female Participants (Study 2). *Note.* Study 2 had participants report stereotypes for singles of their own gender and singles of the other gender. Black words reflect stereotypes by single female participants to describe both single women and single men; red words were stereotypes used to describe only single women; blue words were stereotypes used to only describe single men.

**Figure 4.**
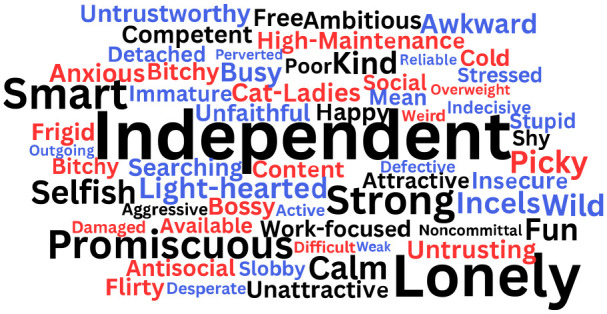
Stereotypes of Single Women and Single Men Provided by Single Male Participants (Study 2). *Note.* Study 2 had participants report stereotypes for singles of their own gender and singles of the other gender. Black words reflect stereotypes used by single male participants to describe both single women and single men; red words were stereotypes used to describe only single women; blue words were stereotypes used to describe only single men.

The stereotypic traits that emerged are illustrated in Figure 2 (Study 1), Figure 3, (Study 2) and Figure 4 (Study 2). Study 1 had participants report stereotypes for singles of their own gender only, while Study 2 had participants report stereotypes for single people of their gender and the other gender. These figures show word clouds with the size of the words depicting the frequency of the stereotype (larger words were mentioned by more participants than smaller words), black words showing common stereotypes that were used to describe both single women and single men, red words showing stereotypes uniquely used to describe single women, and blue words showing stereotypes uniquely used to describe single men. Tables providing the full list of traits and frequencies can be found in the Online Supplemental Materials.

In both studies, the qualitative coding analyses yielded some common stereotypes for single women and single men, that can be understood as *gender-neutral* stereotypes. For example, participants reported that both single women and single men have positive traits, such as being “independent,” “kind,” “smart,” and “free.” Negative gender-neutral stereotypes included being “promiscuous,” “lonely,” “unattractive,” and “selfish.”^5^

Nonetheless, qualitative coding analyses also yielded distinct stereotypes for single women and single men. Single female participants reported that single women are positively stereotyped as “fulfilled,” “resilient” (Study 1), and “lucky” (Study 2) but negatively stereotyped as “high maintenance,” “frigid,” and “bitter” (Studies 1 and 2). In Study 2, single male participants reported unique positive stereotypes of single women as “social,” “fun,” and “content,” and negative stereotypes of single women as, “picky,” “overweight,” and “difficult.”

In Studies 1 and 2, single male participants reported that single men are positively stereotyped as “attractive,” “reliable,” and “light-hearted” (Study 2 only), while negative stereotypes included “slobby,” “immature,” and “stressed” (Study 2 only). Single female participants in Study 2 reported that single men are positively stereotyped as “financially stable” and “carefree” and negatively stereotyped as “players,” “untrustworthy,” and “unfaithful.”

Interestingly, the qualitative analyses also revealed some particularly negative traits about single men and single women. In Study 2, single male participants described a negative stereotype of single men being “misogynistic” and “incels” (*n =* 7; i.e., “involuntarily celibate”: a group of misogynistic men who harass and commit hate crimes against women; O’Malley et al., 2020). Single female participants, did not report stereotypes of single men as incels, but rather single women reported the stereotype of “Mama’s Boys” (i.e., a group of men who remain overly attached to their mothers in adulthood, signaling immaturity and a child-like overreliance on their mothers). In both Studies 1 and 2, single women were described by female and male participants as “Cat Ladies” (*n* = 12; i.e., a single woman who has many cats which she obsesses over).

By reflecting on the overarching themes (Creswell & Poth, 2017), four distinct archetypes of stereotypical single people were identified, with some gendered nuance. For example, the list of positive stereotypes indicated that people tend to think of single people as “independent,” “ambitious,” and “hard-working.” This might be described as, the *Professional* archetype, with *Professional* single women uniquely stereotyped as “successful” and “capable,” and *Professional* single men uniquely stereotyped as “reliable” and “career-driven.” Another group of positive stereotypes included “grounded,” “free,” “kind,” and “fun.” This might represent, the *Carefree* archetype, with *Carefree* single women uniquely stereotyped as “creative” and “open-minded,” and *Carefree* single men uniquely stereotyped as “flexible” and “having free time.” In the list of negative stereotypes, single people were described as “selfish,” “promiscuous,” and “noncommittal.” This might be described as the *Heartless* archetype, with *Heartless* single women uniquely stereotyped as “bitchy” and “untrusting,” and *Heartless* single men uniquely stereotyped as “detached” and “untrustworthy.” Finally, another group of negative stereotypes included “lonely,” “unattractive,” and “antisocial.” This could reflect the *Loner* archetype, with *Loner* single women uniquely stereotyped as “frigid” and “bitter,” and *Loner* single men uniquely stereotyped as “slobby” and “immature.”

## General Discussion

The current research explored, for the first time, whether single women and single men experience differences in discrimination or stereotype content. Table 1 provides a summary of whether hypotheses were supported. Focusing on key findings, the results across two studies highlight that single female and single male participants did *not* differ in the amount of personal discrimination they report experiencing (consistent with the extant quantitative literature; Byrne & Carr, 2005; Conley & Collins, 2002; Greitemeyer, 2009; Hertel et al., 2007; Morris et al., 2007). Reports from single male participants were consistent with the idea that single women and single men do not experience differences in singlism; they reported that single women and single men experience similar amounts of discrimination. In line with this, participants reported similar stereotypic traits for single women *and* single men, which can be understood as four main archetypes: *Professional* (e.g., “independent,” “hard-working”), *Carefree* (e.g., “free,” “fun”), *Heartless* (e.g., “selfish,” “promiscuous”), and *Loner* (e.g., “lonely,” “antisocial”). Yet, despite single female participants reporting similar amounts of personal discrimination and similar stereotype content as single male participants, single female participants believed that single women as a group experience significantly more discrimination than single men as a group (consistent with some qualitative literature Gui, 2020; Pickens & Braun, 2018; Reynolds & Wetherell, 2003 and theorizing Kislev & Marsh, 2023; Lahad, 2013). Although these findings highlight that in many ways, single women and single men experience similar singlism, it also highlights the important gendered nuances that shape perceptions and experiences in singlehood.

### Single Female and Single Male Participants Experience Similar Personal Discrimination

Our research provided novel and robust evidence across both studies that single women and single men report the same amount of singlehood-based discrimination. Specifically, our study is the first (that we are aware of) to directly compare single women and single men’s perceived discrimination ratings. Our findings are consistent with research showing that both single women and single men report experiencing discrimination (Byrne & Carr, 2005; Conley & Collins, 2002; Fisher & Sakaluk, 2020; Girme et al., 2023; Morris et al., 2007), such as being harassed, threatened, disrespected (Byrne & Carr, 2005), given unsolicited advice, and left out of social activities (Girme et al., 2022). Both single women and single men are rated as less agreeable, less attractive, more neurotic (Greitemeyer, 2009), more miserable, lonelier, colder, and less caring than coupled women and men (Hertel et al., 2007). Researchers have posited that because single women violate gender norms for not having a romantic partner and children to care for, they may experience more discrimination than single men (Budgeon, 2008; Gui, 2020; Lahad, 2013; Moore & Radke, 2015; Sharp & Ganong, 2011). Yet, in quantitative studies that have compared ratings of single women and single men’s characteristics, gender differences have been small (Conley & Collins, 2002; Morris et al., 2007). For example, single women were seen as less sexually risky (Conley & Collins, 2002) and more responsible than single men (Morris et al., 2007). Here, we extend existing work by directly demonstrating that single women and single men report similar amounts of personal discrimination.

The qualitative analyses provide further insight about why single women and single men may have reported experiencing similar amounts of discrimination: Several overlapping stereotypes emerged for single women and single men that suggest that overall, they are stereotyped in similar ways. For example, participants described single women and single men with stereotype words consistent with four archetypes: *Professional* (e.g., “independent,” “hard-working,” etc.), *Carefree* (e.g., “grounded,” “fun,” etc.), *Heartless*, (e.g., “promiscuous,” “selfish”), and *Loner*, (e.g., “antisocial,” “lonely,” etc.). When unique words were used to reflect more gender-typical descriptions (e.g., “slut” for women vs. “player” for men), they tended to convey the same underlying stereotypes (e.g., someone who is deemed overly sexually active). An exception to this was in Study 2, in which the words “incels,” “misogynists,” and “perverted” were used to describe single men. These subgroups of men are violent extremists and inflict harm on women and children; single women did not have any equally nefarious stereotypes. Nonetheless, there were some unique words that emerged for single women (e.g., “anxious,” “Cat Lady”) versus single men (e.g., “nerdy,” “Mama’s Boys”) that still fit into the four archetypes identified by the more common stereotypes. Thus, gender norms may shape expectations of what makes someone a “*Loner”;* “Cat Ladies” may be considered *Loners* because they are antisocial, unattractive, and older (or if they are young, they act “older” by foregoing socializing and adventure) while “Mama’s Boys” may be considered *Loners* because they are immature, sensitive, and rely on their mothers’ to meet their needs in adulthood.

### Single Male Participants Perceive Similar Discrimination for Single Women and Single Men

Dovetailing with the similarities in reported personal discrimination, single male participants also reported similar amounts of discrimination against single women and single men, which was also reflected in their ratings of single people in general. One reason for this finding may be because of men’s privileged status in society relative to women (Jost & Kay, 2005; Reuther, 1985; Rudman et al., 2012; Vandello et al., 2008), single men may be unaware of the relevance of gender identity in shaping single people’s experiences, leading them to rate single women and single men similarly (i.e., under-reporting the discrimination against single women). However, another possibility is that due to common media portrayals, stereotypes, and language used to describe single women (e.g., Bridget Jones, “spinster”) and their own personal experiences, single men are aware of the discrimination against both single women and single men (i.e., accurately reporting that single women and single men experience similar amounts of discrimination). Both explanations account for single male participants’ similar ratings of the discrimination experienced by single women and single men and are worth teasing apart in future research.

### Single Female Participants Perceive That Single Women Experience More Discrimination Than Single Men

Although single female and single male participants reported similar amounts of *personal* discrimination, results across both studies demonstrated that single female participants reported that single women as a group experience more discrimination than single men as a group. In addition, single female participants reported higher discrimination toward single people than single male participants. This pattern of reporting lower personal discrimination but higher group discrimination is a long-observed phenomenon for people with marginalized identities (e.g., Bourguignon et al., 2006; Lindsay et al., 2015; Magallares et al., 2016; Taylor et al., 1990). For example, people of color more readily identify racism directed at their racial group compared with racism that they personally encounter (e.g., Bourguignon et al., 2006; Taylor et al., 1990). Similarly, women more readily identify sexism directed at other women compared with themselves (e.g., Bourguignon et al., 2006; Lindsay et al., 2015; Taylor et al., 1990). Thus, single female participants may have been under-reporting their personal discrimination but projecting the discrimination they experience onto single women and single people. Indeed, this phenomenon might explain why qualitative studies demonstrate single women express that they experience a breadth of negative outcomes because of their unique experiences as single women (Addie & Brownlow, 2014; Gui, 2020; Moore & Radke, 2015; Reynolds & Wetherell, 2003). For example, single women may experience more discrimination regarding their sexuality or reproductive choices than single men, as gender expectations for women require a “correct” amount of sexuality, with too little being “prudish” and too much being “promiscuous” (Pickens & Braun, 2018; Reynolds & Wetherell, 2003). These themes also emerged from the qualitative analysis of the current data, with stereotypes such as “frigid” and “promiscuous” illustrating the policing of women’s sexuality.

The discrepancy between personal and group discrimination may serve as a protective mechanism. For example, there is meta-analytic evidence that perceiving oneself to be the subject of discrimination is associated with worse psychological and physical well-being, including greater depression and anxiety, and consuming alcohol or smoking (Pascoe & Richman, 2009; see also Schmitt et al., 2014). In contrast, Bourguignon and colleagues (2006) found that across two studies (with African immigrants and replicated among women), reporting lower personal discrimination and higher discrimination toward one’s group were both associated with higher levels of self-esteem. This may be because under-reporting personal discrimination reduces feelings of personal responsibility for negative experiences while recognizing the discrimination one’s group experiences fosters feelings of connection to their group (Bourguignon et al., 2006; Crocker & Major, 1989). By downplaying instances of personal discrimination, single female participants may be protecting themselves from the consequences of singlism, while simultaneously fostering connection and solidarity with other single women who experience discrimination.

Finally, single female participants might have reported that single women experience more discrimination (despite reporting similar singlism to single male participants) because, ultimately, women face worse *consequences* than men for the same behaviors. There is good evidence for this; female professors are evaluated more negatively in high-status departments compared with low-status departments, indicating that women experience backlash for occupying higher status roles in society (Fisher et al., 2019). Similarly, women in politics are less favored by voters than men (Okimoto & Brescoll, 2010) and women in the military are seen as less skilled than men (Boldry et al., 2001). These biases are concerning because they are indicative that it may be more difficult for women to receive tenure (Deo, 2015; Wilson, 2008), become political leaders (Eagly & Karau, 2002; Rudman et al., 2012), earn the same wage as men (Organisation for Economic Co-operation and Development, 2023; Statistics Canada, 2022), or gain status in other realms that are historically “male-dominated.” However, it is not only high-status women who experience negative bias. Women are burdened with the vast majority of free domestic labor and child care (Chesney & Flood, 2017; Schneider, 2011), and women with longer maternity leaves on their resume are rated as less desirable employees (Hideg et al., 2018; see also Cuddy et al., 2004). Although these examples focus on women’s gender identity, intersectional identities (e.g., race, social class, etc.) have been shown to further exacerbate the discriminatory consequences for women (Berdahl & Moore, 2006; Chaney et al., 2020; Remedios & Snyder, 2018). Thus, trying to reconcile the disconnect between single women and single men’s experiences with singlism involves considering whether single women may experience worse consequences despite being stereotyped in similar ways as single men.

### Strengths and Caveats

By taking a mixed-methods approach, this research helped to integrate qualitative and quantitative findings about the gendered experiences of single women and single men. Furthermore, the current samples were diverse in terms of age (36-83 years) and singlehood histories (e.g., being divorced, widowed, never married, single parents, etc.), which provided more relevant data than the student-based samples commonly used in psychology research. Despite these strengths, this research also has important caveats. Data were collected using Prolific. Prolific data tends to be higher quality data (e.g., participants pass more attention checks, give better open-ended responses, etc.) compared with other online data collection tools such as MTurk, Qualtrics, or SONA (Douglas et al., 2023). Nonetheless, Prolific workers are not representative of the wider population and future research would benefit from gathering diverse samples from ecologically valid community samples.

Furthermore, given that we wanted to maximize participants’ efforts in providing high-quality data for the qualitative reports of singlehood stereotypes, we made decisions to simplify our quantitative assessments of discrimination across different targets (personal, single people, single women, and single men). Although the single-item measures used were adapted based on past research assessing racial and gender-based discrimination (e.g., Taylor et al., 1990), we recognize that these may not be the most reliable measure of singlehood discrimination (see Costello & Osborne, 2019). Indeed, singlehood discrimination may differ from racial and gender-based discrimination because, unlike other forms of discrimination, singlehood-based discrimination is often experienced through interactions with family and friends (e.g., Byrne & Carr, 2005; Girme et al., 2022; Pickens & Braun, 2018) and is evaluated as more acceptable than other forms of discrimination (e.g., Fisher & Sakaluk, 2020; Morris et al., 2007). Future research should consider utilizing more robust multi-item measures of singlehood discrimination to replicate or expand on the existing findings.

Moreover, results may differ in another cultural context, as the samples used in this study were from North America and primarily White. For example, in China, where the marriage and family ideology tend to be stronger, single women have been called “leftover women” and face many social disadvantages relative to single men, such as pressures to put aside their career aspirations to prioritize finding a husband to support his career by caring for him and “his” children (Gui, 2020; see Himawan et al., 2018 for a review). In some Asian countries, it is normative to overtly ask about someone’s age or marital status, and to openly blame someone’s character as the reason they are single, indicating that direct discrimination against singles may be more normative (Himawan et al., 2018). In addition, single women in Turkey are stereotyped as “pure” and “fragile,” indicating that other cultures may see single women as in need of protection and pity (Uğurlu et al., 2021). However, stereotypes of single women in the current research did not strongly reflect “purity” which may be due to cultural differences in gender norms between Turkish and U.S./Canadian participants. Instead, stereotypes of single women in the current literature reflected both negative (e.g., “frigid”; “bitter”) and positive (e.g., “self-aware”; “resilient”) elements of singlehood similar to research conducted using samples from Australia (Addie & Brownlow, 2014) and New Zealand (Reynolds & Wetherell, 2003) indicating that cultural context may be influential in shaping singlehood experiences. Although single people experience singlism cross-culturally, culture may exaggerate the differences in discrimination and stereotype content that single women and single men experience.

Finally, our research only examined the experiences of self-identifying women and men. Although this provided a preliminary examination of discrimination at the intersection of gender and singlehood identities, it does not address the diversity in gender identities. Indeed, gender is largely socialized; it is because of our socialization into roles that women and men may behave, dress, and think differently (e.g., Prentice & Miller, 2006). It is this socialization that shapes experiences of discrimination, which are likely far worse for non-binary and transgender single people because they signal a rejection of the “natural” gender binary and those with multiple systemically oppressed identities often experience worse discrimination (Remedios & Snyder, 2018). However, there is a significant gap in the literature on the experiences of non-binary and transgender people in the context of singlehood (see Girme et al., 2023 and Kislev & Marsh, 2023 for discussions about intersectional identities shaping singlehood outcomes). Future research should evaluate whether or not non-binary and transgender singles experience similar instances of singlism as cisgender singles, and how this impacts their well-being.

## Conclusion

Across two mixed-methods studies employing quantitative and qualitative methods, the results provided novel evidence that single women and single men report similar amounts of personal discrimination and stereotypical content. Nonetheless, single female participants reported that single women as a group experience more discrimination than single men as a group. This research broadens current understandings of single people by illustrating both similarities and discrepancies between single women and single men’s perceptions of discrimination and qualitative insights about the overlapping and unique stereotype content of single women and single men. These findings suggest that single people, regardless of their gender, face stereotyping and discrimination for being single and provide a stepping stone for future work to further examine the potentially distinct consequences of singlism at the intersection of singlehood and gender identity.

## Supplemental Material

sj-docx-1-psp-10.1177_01461672231203123 – Supplemental material for “Cat Ladies” and “Mama’s Boys”: A Mixed-Methods Analysis of the Gendered Discrimination and Stereotypes of Single Women and Single Men Click here for additional data file. Supplemental material, sj-docx-1-psp-10.1177_01461672231203123 for “Cat Ladies” and “Mama’s Boys”: A Mixed-Methods Analysis of the Gendered Discrimination and Stereotypes of Single Women and Single Men by Hannah E. Dupuis and Yuthika U. Girme in Personality and Social Psychology Bulletin

## Notes

1. Participants who indicated they were in a romantic relationship (three cases), failed two or more of the attention check questions (one case), and had empty surveys (six cases) were removed from the analyses, leaving the final sample of 140 participants reported here.

2. Participants who indicated they were in a romantic relationship (two cases) and failed two or more of the attention check questions (two cases) were removed from the analyses, leaving the final sample of 146 participants reported here.

3. Our analyses for H3 to H6 deviated slightly from our pre-registered analytic plan. In Study 1, we had incorrectly pre-registered the analyses for H3 and H4 as a regression, when it should have been a paired samples t-test. In Study 2, we corrected our analytic plan to include paired samples t-tests to test whether single male and single female participants report differences in discrimination against single men versus single women (H3 and H4, see Table 1). However, we did not pre-register our analytic plans for secondary analyses to test whether single participants exhibit in-group bias when reporting discrimination against their in-group (H5 and H6, see Table 1). Thus, the repeated-measures ANOVA and independent samples t-tests were not pre-registered but were required to formally test H5 and H6.

4. We erroneously pre-registered group-mean centering, which is not possible given the cross-sectional design of our studies.

5. The stereotype “selfish” was the most mentioned negative trait word for both single women and single men in Study 1. However, in Study 2, “selfish” was not used by single female participants to describe stereotypes of single women but was used by them for stereotypes of single men. Single male participants used “selfish” as a stereotype for both single women and single men.
