# Supplementary material for: “Cat Ladies” and “Mama’s Boys”: A Mixed-Methods Analysis of the Gendered Discrimination and Stereotypes of Single Women and Single Men
Source: Pers Soc Psychol Bull. 2023 Oct 24;50(2):314–28. doi: 10.1177/01461672231203123 (PMC10860362; doi:10.1177/01461672231203123)
Supplement: sj-docx-1-psp-10.1177_01461672231203123 – Supplemental material for “Cat Ladies” and “Mama’s Boys”: A Mixed-Methods Analysis of the Gendered Discrimination and Stereotypes of Single Women and Single Men [file sj-docx-1-psp-10.1177_01461672231203123.docx]

**ONLINE SUPPLEMENTARY MATERIALS**

**“Cat Ladies” and “Mama’s Boys”: A Mixed-Methods Analysis of the Gendered Discrimination and Stereotypes of Single Women and Men**

[authors blinded for review]

# **Qualitative Frequency Tables.**

## ***OSM Table 1. Frequency of Stereotypical Traits of Single Women and Single Men (Study 1)***

| **Positive Stereotypes** | | | | **Negative Stereotypes** | | | |
| --- | --- | --- | --- | --- | --- | --- | --- |
| **Females’ Stereotypes about Single Women** | **N** | **Males’ Stereotypes about Single Men** | **N** | **Females’ Stereotypes about Single Women** | **N** | **Males’ Stereotypes about Single Men** | **N** |
| Independent* | 70 | Independent | 46 | Selfish | 41 | Selfish | 34 |
| Strong | 28 | Strong | 6 | Lonely | 31 | Lonely | 16 |
| Smart | 24 | Smart | 14 | Difficult | 17 | Difficult | 7 |
| Free | 17 | Free | 26 | Promiscuous | 16 | Promiscuous | 13 |
| Hard-Working | 15 | Hard-Working | 11 | Unattractive | 15 | Unattractive | 7 |
| Confident | 14 | Confident | 8 | Noncommittal | 5 | Noncommittal | 13 |
| Grounded | 13 | Grounded | 6 | Mean | 9 | Mean | 8 |
| Kind | 11 | Kind | 9 | Anti-social | 9 | Anti-social | 6 |
| Financially Stable | 8 | Financially Stable | 10 | Controlling | 24 | Slobby | 13 |
| Fun | 7 | Fun | 6 | Unhappy | 17 | Immature | 10 |
| Happy | 19 | Free Time | 13 | Desperate | 15 | Detached | 9 |
| Social | 13 | Attractive | 6 | Frigid | 15 | Untrustworthy | 8 |
| Fulfilled | 11 | Reliable | 5 | Bitter | 15 | Substance Abusing | 8 |
| Resilient | 11 | Career Driven | 5 | Unstable | 12 | Irresponsible | 7 |
| Capable | 9 | Flexible | 4 | High-Maintenance | 12 | Poor | 6 |
| Self-aware | 9 |  |  | Nitpicky | 8 | Angry | 6 |
| Adventurous | 7 |  |  | Stubborn | 8 | Defective | 5 |
| Courageous | 6 |  |  | Boring | 7 | Socially Awkward | 5 |
| Creative | 6 |  |  | Untrusting | 5 | Weird | 4 |
| Trustworthy | 6 |  |  | Cruel | 5 | Nerdy | 4 |
| Successful | 5 |  |  | Cat Lady | 5 | Clingy | 4 |
| Open-Minded | 4 |  |  | Fragile | 4 |  |  |
|  |  |  |  | Independent* | 4 |  |  |
|  | **313** |  | **175** |  | **299** |  | **193** |

## ***OSM Table 2. Frequency of Stereotypical Traits of Single Women and Single Men (Study 2)***

| **Females’ Stereotypes** | | | | **Males’ Stereotypes** | | | |
| --- | --- | --- | --- | --- | --- | --- | --- |
| **Single Women** | **N** | **Single Men** | **N** | **Single Women** | **N** | **Single Men** | **N** |
| Lonely | 43 | Lonely | 32 | Lonely | 29 | Lonely | 47 |
| Independent | 60 | Independent | 32 | Independent | 24 | Independent | 23 |
| Smart | 20 | Smart | 8 | Smart | 6 | Smart | 10 |
| Kind | 11 | Kind | 8 | Kind | 10 | Kind | 4 |
| Poor | 10 | Poor | 6 | Poor | 5 | Poor | 6 |
| Ambitious | 9 | Ambitious | 17 | Ambitious | 8 | Ambitious | 13 |
| Unattractive | 9 | Unattractive | 10 | Unattractive | 16 | Unattractive | 9 |
| Attractive | 8 | Attractive | 5 | Attractive | 6 | Attractive | 5 |
| Promiscuous | 7 | Promiscuous | 26 | Promiscuous | 5 | Promiscuous | 19 |
| Aggressive | 4 | Aggressive | 8 | Aggressive | 5 | Aggressive | 10 |
| Shy | 4 | Shy | 8 | Shy | 11 | Shy | 20 |
| Strong | 21 | Selfish | 28 | Free | 18 | Sad | 26 |
| Happy | 19 | Work-focused | 14 | Picky | 14 | Free | 26 |
| Sad | 19 | Untrustworthy | 13 | Sad | 14 | Happy | 16 |
| Free | 18 | Noncommittal | 12 | Social | 13 | Selfish | 15 |
| Damaged | 15 | Detached | 11 | Difficult | 11 | Weird | 14 |
| Picky | 14 | Confident | 10 | Selfish | 11 | Slobby | 13 |
| Confident | 13 | Immature | 9 | Fun | 10 | Light-hearted | 13 |
| Desperate | 13 | Financially Stable | 9 | Strong | 10 | Defective | 12 |
| Work-focused | 12 | Happy | 9 | Anxious | 8 | Untrustworthy | 11 |
| Spinster | 11 | Picky | 8 | Flirty | 8 | Adventurous | 10 |
| Difficult | 10 | Slobby | 8 | Bossy | 8 | Lazy | 10 |
| Hard-working | 9 | Fun | 8 | Available | 7 | Irresponsible | 10 |
| Stubborn | 8 | Social | 7 | Defective | 7 | Financially Stable | 9 |
| Confident | 7 | Carefree | 6 | Weird | 6 | Available | 9 |
| Cat Ladies | 7 | Insecure | 5 | Bitchy | 6 | Work-focused | 9 |
| Fun | 7 | Mama’s Boys | 5 | Work-focused | 6 | Desperate | 8 |
| Bitter | 6 | Desperate | 5 | Cold | 6 | Boring | 8 |
| Frigid | 6 | Unfaithful | 4 | High maintenance | 5 | Stressed | 7 |
| Bitchy | 5 | Stupid | 4 | Overweight | 5 | Incels | 7 |
| Interesting | 5 | Indecisive | 4 | Adventurous | 5 | Wild | 7 |
| Lucky | 5 | Defective | 4 | Frigid | 4 | Noncommittal | 7 |
| Weird | 5 | Interesting | 4 | Cat Ladies | 4 | Weak | 6 |
| Competent | 4 | Searching | 4 | Content | 4 | Outgoing | 6 |
| Untrusting | 4 | Awkward | 4 |  |  | Awkward | 6 |
| Cold | 4 | Busy | 4 |  |  | Immature | 6 |
| Calm | 4 |  |  |  |  | Confident | 6 |
| Anxious | 4 |  |  |  |  | Perverted | 5 |
| Social | 4 |  |  |  |  | Strong | 5 |
| High maintenance | 4 |  |  |  |  | Calm | 5 |
| Lazy | 4 |  |  |  |  | Mean | 4 |
| Crazy | 4 |  |  |  |  | Reliable | 4 |
| Antisocial | 4 |  |  |  |  | Active | 4 |
|  | 454 |  | 366 |  | 315 |  | 473 |
